# The 3 Wishes Program Improves Families’ Experience of Emotional and Spiritual Support at the End of Life

**DOI:** 10.1007/s11606-022-07638-7

**Published:** 2022-05-17

**Authors:** Thanh H. Neville, Zachary Taich, Anne M. Walling, Danielle Bear, Deborah J. Cook, Chi-Hong Tseng, Neil S. Wenger

**Affiliations:** 1 Division of Pulmonary, Critical Care, and Sleep Medicine, Department of Medicine, David Geffen School of Medicine, UCLA, Los Angeles, CA USA; 2 Division of General Internal Medicine and Health Services Research, Department of Medicine, David Geffen School of Medicine, UCLA, Los Angeles, CA USA; 3 VA Greater Los Angeles Healthcare System, Veteran Affairs, Los Angeles, USA; 4 UCLA Office of the Patient Experience, UCLA Health, Los Angeles, CA USA; 5 Department of Medicine, Faculty of Health Sciences, McMaster University, Hamilton, Ontario Canada

**Keywords:** End of life, Palliative care, Intensive care unit

## Abstract

**Background:**

The end-of-life (EOL) experience in the intensive care unit (ICU) is emotionally challenging, and there are opportunities for improvement. The 3 Wishes Program (3WP) promotes the dignity of dying patients and their families by eliciting and implementing wishes at the EOL.

**Aim:**

To assess whether the 3WP is associated with improved ratings of EOL care.

**Program Description:**

In the 3WP, clinicians elicit and fulfill simple wishes for dying patients and their families.

**Setting:**

2-hospital academic healthcare system.

**Participants:**

Dying patients in the ICU and their families.

**Program Evaluation:**

A modified Bereaved Family Survey (BFS), a validated tool for measuring EOL care quality, was completed by families of ICU decedents approximately 3 months after death. We compared patients whose care involved the 3WP to those who did not using three BFS–derived measures: Respectful Care and Communication (5 questions), Emotional and Spiritual Support (3 questions), and the BFS-Performance Measure (BFS-PM, a single-item global measure of care).

**Results:**

Of 314 completed surveys, 117 were for patients whose care included the 3WP. Bereaved families of 3WP patients rated the Emotional and Spiritual Support factor significantly higher (7.5 vs. 6.0, *p* = 0.003, adjusted *p* = 0.001) than those who did not receive the 3WP. The Respectful Care and Communication factor and BFS-PM were no different between groups.

**Discussion:**

The 3WP is a low-cost intervention that may be a feasible strategy for improving the EOL experience.

## INTRODUCTION

End-of-life (EOL) care is a crucial part of patient and family-centered care in the intensive care unit (ICU). Unfortunately, a family’s experience of an ICU admission that ends in their loved one’s death has been likened to a “vortex” in which families are confronted by life-support machines, technical terminology, and professional strangers as they witness the downward spiral of their loved one’s health.^1^ As such, families of patients who die in the ICU often suffer from depression,^2–5^ post-traumatic stress disorder (PTSD),^3–5^ complicated grief,^6^ and reduced quality of life.^7^

Although there is recognition that EOL care in the ICU needs to be improved,^8,9^ several interventions have failed to improve families’ assessments of their loved ones’ EOL care.^10–13^ In a systematic review of 14 studies that evaluated factors associated with family satisfaction with EOL care in the ICU, only 3 were interventions that showed improvement in the quality of EOL care and only one used a validated instrument to evaluate the outcome of interest.^14^ Furthermore, many studies focus on measuring improvements in processes of care (i.e., limitations of life-sustaining treatments, length of stay),^15^ and while these changes are important, they do not necessarily reflect patient and family perceptions.

The 3 Wishes Program (3WP) postulates that the elicitation and implementation of small wishes for dying patients and their families by healthcare workers (HCWs) can improve the EOL experience for patients and their families.^16,17^ Examples of wishes include playing the patient’s favorite music, providing a non-hospital blanket, orchestrating a final “date night,” and providing grieving family members with keepsakes. In qualitative evaluations of focus groups and semi-structured interviews, the 3WP has been shown to enrich interpersonal connections; ease family grief; celebrate legacies; and enhance clinician satisfaction.^16–23^ However, larger-scale, empirical evaluation of the 3WP using a standardized instrument is lacking.

In this evaluation, we used a modified version of the Bereaved Family Survey (BFS) to compare family ratings of EOL care for patients whose care involved the 3WP versus those who did not. We hypothesized that the 3WP will be associated with higher ratings of emotional and spiritual support.

## SETTING AND PARTICIPANTS

The 3WP was implemented in 6 adult ICUs of a 2-hospital academic healthcare system. HCWs implemented wishes for dying patients and their families (note: in this manuscript, we use the word “family” to refer to the patient’s closest loved ones). Weekly lists of all patients who died in these ICUs were obtained from the electronic health record (EHR) between July 2019 and April 2021. These lists were shared with Risk Management to avoid mailing surveys to family members who might be adversely affected by the survey. Family addresses were obtained by chart review, and if unavailable, the research assistant called the listed emergency contact to confirm the appropriate surrogate and obtain the address.

## PROGRAM DESCRIPTION

Patients were eligible for the 3WP once a decision to withdraw life support was made or the healthcare team agreed that the patient’s probability of dying in the hospital was > 95%. As a quality-improvement intervention, 3WP initiation was at the discretion of the clinical team and thus may not be offered to all patients who met eligibility. Patients and/or families provided verbal consent. HCWs (mostly nurses) asked how they might bring comfort to a dying patient and their family in the final hours or days of life. Wishes were implemented by HCWs, sometimes with the help of the 3WP project manager. During the COVID-19 pandemic when many families could not be at the bedside, HCWs were encouraged to reach out to the 3WP project manager to offer keepsakes via mail.

The 3WP was initiated in the Medical ICU of the studied health system in December 2017 and subsequently expanded to all six adult ICUs (medical ICU, neurocritical care unit, cardiac care unit, cardiothoracic ICU, liver transplant ICU, and an academic community hospital mixed-use ICU) by November 2020. Prior to launch in each unit, at least two nurse champions were identified and trained to serve as “on-the-ground” leaders for the unit. Each unit was supplied with an inventory of commonly used supplies (i.e., fingerprint keepsakes, blankets, frames, etc.) and received the assistance of a project manager, if needed.

## PROGRAM EVALUATION

The survey, a cover letter, and a self-addressed stamped envelope were mailed to the family 3 months after the patient’s death. Surveys had no identifying information and were tracked using a unique code. No incentive was offered. Two weeks following the initial mailing, non-responders received up to three telephone calls (at least one attempt after 5 p.m.) to request the return of the survey or to complete it by telephone, if preferred. Non-English speakers and patients without documented family were excluded. Survey responses were stored on REDCap.^24^ As a quality-improvement initiative that was launched at staggered dates over time, the 3WP was more established in some units than others at the time of survey administration. Patient demographics, insurance, highest Sequential Organ Failure Assessment (SOFA)^25^ score within the first 24 h of ICU admission, type of ICU in which the patient died, whether the patient received a palliative care consult, COVID-19 infection, and whether the patient required vasopressors, mechanical ventilation, and dialysis were extracted from the EHR. Presence of advance care planning documents (advance directive, power of attorney for healthcare, or POLST form) was also noted.

### Modified Bereaved Family Survey

The BFS is a National Quality Forum–endorsed instrument that asks family members to rate specific and global aspects of care that a deceased veteran received from the Veterans Administration (VA) in the last month of life.^26–29^ Our modified version includes 13 forced-choice items and excludes 5 questions concerning veteran death benefits.

Individual items were analyzed by dichotomizing the 5-point Liker scale into “excellent” versus all other responses (scores reflect the percentage of respondents who answered “excellent”). Additionally, 3 BFS–derived measures were analyzed: Respectful Care and Communication (5 questions, alpha = 0.82), Emotional and Spiritual Support (3 questions, alpha = 0.77), and the BFS-Performance Measure (BFS-PM, a single item asking for a global rating of EOL care). The Respectful Care and Communication factor score sums five items about staff behavior (total ranges from 0 to 15): (1) listened to concerns; (2) provided medical treatment patient wanted; (3) were kind, caring, and respectful; (4) kept family members informed about patient’s condition and treatment; and (5) attended to personal care needs. The Emotional and Spiritual Support factor score sums three items about whether staff provided (total ranges 0–9): (1) enough emotional support before death; (2) enough spiritual support; and (3) enough emotional support after death. We hypothesized that the Emotional and Spiritual Support Factor score would be most responsive to the 3WP intervention.

Three additional survey questions queried whether families felt the patient died in the right place (from Views of Informal Carers – Evaluation of Services)^30^, whether the hospital worked well with the patient’s continuity providers, and whether there was unwanted treatment (from National Health and Aging Trends Study).^31,32^

### Statistical Analysis

Descriptive statistics are displayed as proportions. Differences between study groups were assessed using a Wilcoxon rank sum for continuous variables and chi-square test for categorical variables. For each outcome, including all individual questions and factor scores, a multivariate regression model was fitted to adjust for age, gender, marital status, respondent’s relationship to patient, race/ethnicity, primary language, type of insurance, type of ICU, presence of COVID-19 infection, palliative care consultation, presence of an advance directive or POLST, and need for mechanical ventilation, vasopressors, or dialysis. Statistical analyses were performed using RStudio statistical software (Version 1.4.1717).

## RESULTS

**Fig. 1:**
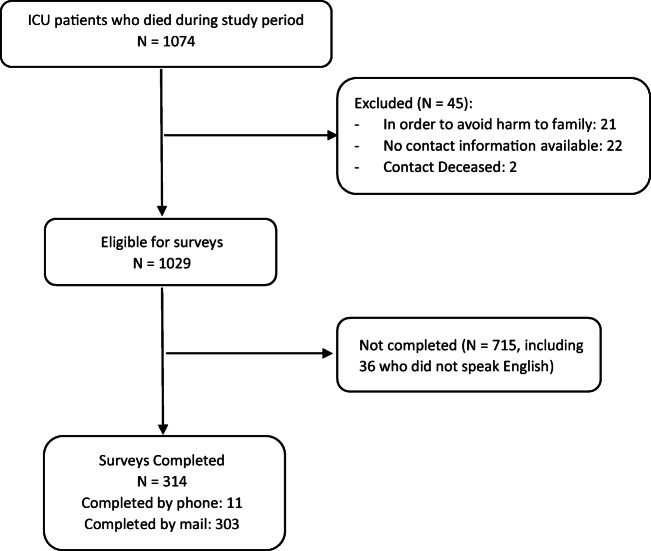
Flowchart of study sample.

During the study period, 1074 adults died in the ICUs of the health system (Fig. 1). Of these, 45 were excluded (21 were excluded by the Risk Management for potential harm to family, 22 had no contact information, and 2 had contacts that were deceased). Of the 1029 mailed surveys, 314 (30.5%) were completed (of which, 117 surveys were from families whose loved ones’ EOL care involved the 3WP).

Table 1 shows bivariate comparisons between patients whose families returned surveys and those who did not, and between those who had 3WP incorporated into their EOL care versus those who did not. In comparison to non-respondents, the patients for whom families completed surveys were older, more often non-Hispanic white, and more often English-speaking; had an advance care planning document in the EHR; and were less likely to have needed vasopressors or dialysis. Among those for whom surveys were received, decedents whose care involved the 3WP were younger, less often male, and less often non-Hispanic white; had a longer length of stay; and were more likely to have died during a COVID–related hospitalization.

**Table 1:** Patient Demographics and Clinical Characteristics

Characteristic	All ICU decedents			ICU decedents with completed surveys		
	Survey not completed (*N* = 715)	Survey completed (*N* = 314)	*p* value	3WP (*N* = 117)	Usual care (*N* = 197)	*p* value
Patient whose care included the 3WP, *N* (%)	264 (36.9%)	117 (37.3%)	0.918			
Age at death (years), median (IQR)	66 (53–77)	69 (59–78)	0.002	69 (57–75)	70 (61–79)	0.024
Gender, female, *N* (%)	320 (44.8%)	147 (46.8%)	0.480	64 (54.7%)	83 (42.1%)	0.031
Marital status, married, *N* (%)	385 (53.8%)	179 (57.0%)	0.348	72 (61.5%)	107 (54.3%)	0.211
Race/ethnicity, non-Hispanic white, *N* (%)	269 (38.1%)	160 (51.3%)	< 0.001	44 (37.6%)	116 (59.5%)	< 0.001
Language, English, *N* (%)	564 (78.9%)	269 (85.7%)	0.011	96 (82.1%)	173 (87.8%)	0.159
Insurance, public, *N* (%)	508 (71.0%)	218 (69.4%)	0.599	76 (65.0%)	142 (72.1%)	0.185
ICU			0.135			< 0.001
Mixed surgical and medical ICU, *N* (%)	197 (27.6%)	80 (25.5%)		26 (22.2%)	54 (27.4%)	
Medical ICU, *N* (%)	216 (30.2%)	95 (30.3%)		57 (48.7%)	38 (19.3%)	
Neurocritical care unit, *N* (%)	74 (10.3%)	38 (12.1%)		6 (5.1%)	32 (16.2%)	
Cardiac care unit, *N* (%)	38 (5.3%)	29 (9.2%)		0 (0.0%)	29 (14.7%)	
Cardiothoracic ICU, *N* (%)	83 (11.6%)	26 (8.3%)		15 (12.8%)	11 (5.6%)	
Liver transplant surgical ICU, *N* (%)	107 (15.0%)	46 (14.6%)		13 (11.1%)	33 (16.8%)	
COVID-19 infection, *N* (%)	112 (15.7%)	43 (13.7%)	0.416	31 (26.5%)	12 (6.1%)	< 0.001
Hospital length of stay (days), median (IQR)	9 (3–19)	9 (3–21)	0.488	14 (5–25)	7 (2–17)	< 0.001
SOFA on ICU admission, median (IQR)	7 (4–9)	7 (4–9)	0.783	8 (4–9)	5 (4–9)	0.061
Palliative care consultation, *N* (%)	252 (35.2%)	104 (33.1%)	0.510	34 (29.1%)	70 (35.5%)	0.239
Advance directive or POLST document available in EHR, *N* (%)	201 (28.1%)	113 (36.0%)	0.012	38 (32.5%)	75 (38.1%)	0.318
Mechanical ventilation, *N* (%)	562 (78.6%)	241 (76.8%)	0.509	91 (77.8%)	150 (76.1%)	0.740
Vasopressors, *N* (%)	524 (73.3%)	211 (67.2%)	0.046	61 (52.1%)	150 (76.1%)	< 0.001
Dialysis, *N* (%)	124 (17.3%)	37 (11.8%)	0.024	12 (10.3%)	25 (12.7%)	0.518

*ICU* intensive care unit, *3WP* 3 Wishes Program, *IQR* interquartile range, *SOFA* sequential organ failure assessment, *POLST* Physician Orders for Life Sustaining Treatment, *EHR* electronic health record

### Patients and Wishes

The 3WP was incorporated into the EOL care of 386 (36%) patients among the 1074 ICU patients who died during the study period. Among ICU decedents for whom surveys were received, a nearly identical proportion (117/314 = 37%) received the 3WP as part of their EOL care. For these 117 patients, ICU staff fulfilled 389 wishes at a mean cost of $27 per patient. Half of all wishes (193) were keepsakes, created and given to bereaved family members by HCWs (Table 2). These included fingerprint keychains, locks of hair, framed EKGs, hand sculptures, and fingerprint paintings. Other common wishes include decorating the room (19% of total wishes) and providing music during final moments (9% of total wishes). The 3WP was initiated and implemented by ICU nurses in most cases (77%).

**Table 2:** 3 Wishes Program Characteristics for Survey Respondents

3WP initiator, *N* (%)	*N* = 117
Bedside nurse	90 (77%)
3 wishes team, not part of clinical team	15 (13%)
ICU physician	8 (7%)
Social work	2 (2%)
Palliative care	1 (1%)
Spiritual care	1 (1%)
Wish category, *N* (%)	*N* = 389
Keepsakes	193 (50%)
Humanizing the environment*	74 (19%)
Music	36 (9%)
Word clouds	25 (6%)
Rituals and spiritual support	18 (5%)
Facilitating connections	14 (4%)
Providing food and beverages for family	9 (2%)
Family care	6 (2%)
Treating the patient as an individual^†^	6 (2%)
Preparations and final arrangements	4 (1%)
Other	4 (1%)
Wish implemented by, *N* (%)	*N* = 389
Bedside ICU nurse	299 (77%)
> 1 person	39 (10%)
3 wishes team	32 (8%)
Spiritual care	9 (2%)
Other	10 (1%)
Timing of wish implementation, *N* (%)	*N* = 389
Antemortem	310 (80%)
Postmortem	79 (20%)

*Examples include hanging up pictures depicting the patient’s favorite things, providing the patient with a non-hospital blanket, putting up special occasion decorations (holiday, anniversary, birthday, wedding, etc.), buying the patient’s favorite flowers for the room

^**†**^Examples include allowing the patient to wear clothes from home, pampering the patient with spa day, allowing the patient to go outside, swabbing the patient’s mouth with their favorite food or beverage

### Ratings of End-of-Life Care

In bivariate analysis, families of patients who received 3WP as part of their EOL care were more likely to answer “always” to questions asking how often they were kept informed, how often they felt spiritually supported, and how often they felt emotionally supported during the patient’s last month of life (Table 3). They were less likely to believe that their loved one died in the right place, although this was no longer significant after adjusting for covariates. After adjustment for covariates, families of patients who received the 3WP as part of their EOL care, compared to those without the 3WP, were more likely to respond with “always” to the following: how often they were kept informed about the patient’s condition and treatment (OR = 2.5), how often they felt emotionally supported in the last month of life (OR = 2.5), and how often they felt emotionally supported after the patient’s death (OR = 2.7).

**Table 3:** End-of-Life Care Survey Outcomes Comparing Patients Who Received or Did Not Receive the 3 Wishes Program

	Number of respondents agreeing, *N* (%)			Adjusted 3WP vs usual care odds ratio	
	3WP (*N* = 117)	Usual care (*N* = 197)	*p* value	Adjusted value*	*p* value
Bereaved family survey items (regarding last month of life)				Odds ratio (95% CI)	
Respectful care and communication					
Staff always took the time to listen	90 (78.3%)	135 (72.6%)	0.270	1.70 (0.87 to 3.40)	0.127
Staff always provided treatment the patient wanted	86 (74.8%)	134 (72.4%)	0.654	1.29 (0.66 to 2.54)	0.464
Staff were always kind, caring, and respectful	96 (83.5%)	154 (81.1%)	0.593	1.48 (0.68 to 3.27)	0.327
Staff always kept family informed about the patient’s condition and treatment	87 (75.7%)	118 (62.8%)	0.020	2.47 (1.30 to 4.83)	0.006
Staff always attended to the patient’s personal care needs (bathing, dressing, eating meals)	76 (66.1%)	120 (65.2%)	0.878	1.22 (0.66 to 2.31)	0.529
Care around time of death					
Staff alerted the family that the patient was about to die	100 (87.7%)	170 (88.5%)	0.829	0.64 (0.26 to 1.59)	0.337
Management of symptoms					
Patient did not experience pain	37 (33.0%)	70 (38.9%)	0.313	0.89 (0.46 to 1.70)	0.722
Pain never made patient uncomfortable	23 (26.1%)	33 (24.3%)	0.752	1.01 (0.43 to 2.35)	0.986
Emotional and spiritual support					
Staff always provided family/patient with spiritual support	60 (54.5%)	72 (40.4%)	0.020	1.79 (0.98 to 3.30)	0.061
Staff always provided family/patient with emotional support	74 (65.5%)	95 (51.9%)	0.022	2.52 (1.37 to 4.75)	0.003
Staff always provided family with emotional support after patient’s death	73 (65.2%)	102 (54.3%)	0.063	2.70 (1.44 to 5.22)	0.002
Performance measure					
Excellent overall rating of care received during last month of life	79 (69.3%)	119 (63.0%)	0.262	1.48 (0.79 to 2.82)	0.220
Additional questions					
Patient died in the right place	78 (69.6%)	159 (81.5%)	0.017	0.75 (0.36 to 1.59)	0.457
Hospital definitely worked well with primary care and other providers	51 (45.5%)	100 (53.2%)	0.200	1.63 (0.90 to 2.97)	0.107
No decisions were made that the patient would not have wanted	90 (78.3%)	151 (79.9%)	0.733	0.77 (0.38 to 1.57)	0.470
BFS factor scores	Unadjusted median (IQR)			*β* coefficient*	
Respectful care and communication factor score (*N* = 284)	14.0 (12 to 15)	14.0 (11 to 15)	0.225	0.89 (− 0.04 to 1.83)	0.063
Emotional and spiritual support factor score (*N* = 277)	7.5 (6–9)	6.0 (3–9)	0.003	1.37 (0.54 to 2.19)	0.001

Missing data for survey questions ranged from 0 to 8.3%

*Adjust for age, gender, marital status, respondent’s relationship to patient, race/ethnicity, primary language, type of insurance, type of ICU, presence of COVID-19 infection, palliative care consultation during hospitalization, presence of an advance directive or POLST, and need for mechanical ventilation, vasopressors, and dialysis

Emotional and Spiritual Support factor scores were higher in the group that received 3WP, compared to patients who did not (adjusted mean 6.66 vs. 5.30, *p* = .001). There was no significant difference in the BFS-PM (excellent 69.3% vs 63.0%, *p* = 0.26) or the Respectful Care and Communication factor (adjusted mean 13.1 vs. 12.2, *p* = .063) between the two groups.

## DISCUSSION

Among patients dying in the ICU, receipt of wishes through the 3WP was associated with significantly better family ratings of emotional and spiritual support at the EOL in this quality-improvement initiative. The finding that the 3WP moved the needle in improving BFS ratings, which asks about their loved one’s experience during the last *month* of life, underscores the crucial importance and lasting impression of final moments. In contrast, elaborate, costly, and time-consuming interventions, such as multi-faceted efforts to improve EOL care in the ICU, have not demonstrated improvements in family-assessed quality of dying or in family satisfaction with care.^10,12^ The 3WP is inexpensive, often occurs in the last hours to days of life, and carries minimal risk. It has also been shown to be adaptable and sustainable even during the extraordinary circumstances of the COVID-19 pandemic.^33^

It is important to recognize that the premise of the 3WP is broad—it is an umbrella term used for acts of kindness implemented or facilitated by HCWs for their dying patients and their families. For example, a bedside clinician might elicit that the patient and/or family can benefit from a visit from spiritual care or social work and thus facilitate a visit—both of these services have been shown to be associated with increased family satisfaction with ICU care.^34,35^ Also, simply by asking and giving permission, the 3WP also empowers families to carry out their own ideas that might comfort the patient (i.e., bring items from home). Nonetheless, the majority of wishes implemented in this study (such as providing keepsakes or decorating the hospital room) are unique to the program and would not have been implemented otherwise. Keepsakes, which we have previously demonstrated to foster comfort and meaning for families,^19^ were half of all wishes implemented during this study, and may also play a role in the higher ratings for emotional support *after* death.

This observational study is not without limitations. As a quality-improvement study and not a randomized control trial, patients were not randomized and we cannot exclude the possibility of selection bias. The 3WP is a unit- and institution-level intervention^23^ such that randomization of individual patients could increase the risk of contamination in that the 3WP intervention would likely be used in the control group. We did not collect data on the HCWs implementing the 3WP or other systemic factors that may influence whether and why some patients experienced the 3WP as part of their EOL care and others did not. We are also unable to adjust for respondent characteristics other than their relationship to the patient, but this is similar to prior studies using the BFS.^36,37^

This study was performed in a single healthcare system, and it is plausible that similar BFS results would not be found at other institutions that may have different resources for the 3WP. However, the 3WP has been implemented in multi-center studies,^18,19,23^ including community hospitals.^38,39^ Although our response rate was low (30%), this is not uncommon for studies evaluating the quality of dying that are outside the integrated VA system.^40–42^

Although a simple concept, the 3WP seeks to change the perspective around death, such that dying is also seen as an opportunity to honor a patient’s wishes, celebrate the patient’s life, and create peaceful memories during final moments. Wishes implemented in the program emphasize life review, memory making, and the affirmation and celebration of the patient as a person, which have all been described as important components of a “good death.”^43–45^ The 3WP is individualized and caters to the non-medical needs of a patient and their family. Furthermore, it is novel in that it is an example of primary palliative care and implemented by bedside ICU clinicians. Even though it is only a brief slice of the ICU experience, the 3WP reframes the interaction between clinicians and patients from treating to nurturing. Family ratings of care suggest that this reframing may recast the family’s ICU experience.
